# Component-Resolved Diagnosis of Hazelnut Allergy in Children

**DOI:** 10.3390/nu13020640

**Published:** 2021-02-16

**Authors:** Carlo Caffarelli, Carla Mastrorilli, Angelica Santoro, Massimo Criscione, Michela Procaccianti

**Affiliations:** 1 Clinica Pediatrica, Dipartimento di Medicina e Chirurgia, Università di Parma, Azienda Ospedaliero-Universitaria di Parma, 43126 Parma, Italy; angelica.santoro204@gmail.com (A.S.); michela.procaccianti@outlook.it (M.P.); 2 UO Pediatria e Pronto Soccorso, Azienda Ospedaliero-Universitaria Consorziale Policlinico, Ospedale Pediatrico Giovanni XXIII, 70126 Bari, Italy; carla.mastrorilli@icloud.com; 3 Dipartimento di Medicina e Chirurgia, Università di Parma, Azienda Ospedaliero-Universitaria di Parma, 43126 Parma, Italy; massimo.criscione@studenti.unipr.it

**Keywords:** hazelnut, hypersensitivity, component-resolved diagnostics, children, food allergy, Cor a 1, Cor a 14, Cor a 9, IgE

## Abstract

Hazelnuts commonly elicit allergic reactions starting from childhood and adolescence, with a rare resolution over time. The definite diagnosis of a hazelnut allergy relies on an oral food challenge. The role of component resolved diagnostics in reducing the need for oral food challenges in the diagnosis of hazelnut allergies is still debated. Therefore, three electronic databases were systematically searched for studies on the diagnostic accuracy of specific-IgE (sIgE) on hazelnut proteins for identifying children with a hazelnut allergy. Studies regarding IgE testing on at least one hazelnut allergen component in children whose final diagnosis was determined by oral food challenges or a suggestive history of serious symptoms due to a hazelnut allergy were included. Study quality was assessed by the Quality Assessment of Diagnostic Accuracy Studies-2 tool. Eight studies enrolling 757 children, were identified. Overall, sensitivity, specificity, area under the curve and diagnostic odd ratio of Cor a 1 sIgE were lower than those of Cor a 9 and Cor a 14 sIge. When the test results were positive, the post-test probability of a hazelnut allergy was 34% for Cor a 1 sIgE, 60% for Cor a9 sIgE and 73% for Cor a 14 sIgE. When the test results were negative, the post-test probability of a hazelnut allergy was 55% for Cor a 1 sIgE, 16% for Cor a9 sIgE and 14% for Cor a 14 sIgE. Measurement of IgE levels to Cor a 9 and Cor a 14 might have the potential to improve specificity in detecting clinically tolerant children among hazelnut-sensitized ones, reducing the need to perform oral food challenges.

## 1. Introduction

Corylus avellana belongs to the same tree family of alders and birches (Betulaceae). Hazelnut is recognized as a common nut triggering allergic reactions from childhood and adolescence, and its prevalence varies by region. The self-reported prevalence of hazelnut allergies is approximately 0.2% in children [1] and up to 4.5% among adults from birch-endemic areas [2]. Resolution of a hazelnut allergy is rare (9% of cases), and children tend to have the disease for their whole life [3]. Clinical presentation differs from age [4]. Hazelnut allergies are associated with severe reactions in childhood and are one of the most common causes of anaphylactic death in adolescents and young adults [5]. On the contrary, adults mainly experience localized oral symptoms due to cross-reactions with pollens, in particular birch and alder. 

Current management in childhood is based on a strict elimination diet, along with education of patients, families, and caregivers on managing allergic reactions caused by accidental ingestion [6], which is frequent in allergic individuals [7,8]. Since hazelnut is widespread in many processed foods and bakery products (especially pastries and chocolates), the dietary restriction and the constant fear of a severe reaction significantly worsen the quality of life of affected patients and their families. Therefore, it is essential to correctly diagnose hazelnut allergy with the aim of avoiding unnecessary therapeutic measures that limit the patient’s quality of life. Hazelnut allergies are diagnosed with a combination of a convincing clinical history, serum-specific IgE (sIgE), skin prick testing (SPT), and oral food challenges (OFCs) [6,9,10,11,12]. Extract-based hazelnut tests (SPT and sIgE) have high sensitivity but low specificity (6-28% for SPT, using respectively natural and commercial extracts; 17–77% for sIgE, depending on the cutoff) [13] due to cross-sensitization with pollen or other food allergens that present high homology with the allergen tested. IgE sensitization to hazelnut extract is common, especially in birch endemic areas, it can occur whether patients react to hazelnut with severe or mild symptoms or even if no reaction occurs, and it often requires an OFC to assess the clinical significance [4,14,15,16,17]. Raising the cut-off values does not increase the sensitivity of SPT and sIgE [18]. OFC is considered the gold standard for diagnosis [19], even if it is an expensive, time-consuming test, with the risk for the patient of potentially life-threatening allergic reactions. 

Component-resolved diagnostics (CRDs) has been introduced in clinical practice to more accurately discriminate patients who are not only sensitized to hazelnut but also allergic, and it is becoming an essential tool able to improve diagnostic accuracy [20].

The allergens of hazelnut belong to the families of seed storage proteins, pathogenesis-related proteins (PR-10), lipid transfer proteins (LTP), profillin and oleosine.

Genuine allergies to hazelnuts are generally due to sensitization to storage proteins or LPTs in children. Storage proteins are heat-stable and resistant to gastric digestion. They are well represented in hazelnuts and may account for more severe reactions, in particular Cor a 9, 11S globulin; and Cor a 14, 2s albumin. Also, LTPs (Cor a 8) are resistant to heat and digestion and are correlated with more serious symptoms. [15,17,21]. The pathogenesis-related class 10 proteins (PR-10) belong to one of the 11 subfamilies of the Bet v 1 family. Cross-reactive allergy to hazelnuts develops in birch pollen allergic individuals sensitized to Bet v 1 (PR-10).

Hazelnut contains Cor a 1, a PR-10 labile to heat and digestion that is a highly cross-reactive allergen shared with the main birch protein, Bet v 1. Cor a 1 was introduced in 2007 by one of the commercial producers of a serum hazelnut-specific sIgE test to improve the test’s sensitivity for birch-related reactions to hazelnut, but it resulted in positive tests without clinical relevance [20]. Patients with an allergy to pollen birch generally develop mild to moderate symptoms. Clinical relevance of other hazelnut components, such as the 7S-vicilinlike protein Cor a 11 and two oleosins (Cor a 12 and Cor a 13), has not been confirmed [22]. The aim of this systematic review was to assess the diagnostic accuracy of sIgE on individual hazelnut proteins in the diagnostic work-up of hazelnut allergies in children.

## 2. Materials and Methods

We systematically searched three key electronic databases: MEDLINE (Pubmed), EMBASE (Ovid) and the Cochrane library. The databases were searched from 2010 to February 28th, 2020, using the search terms: “IgE”, “prick”, “SPT”, “diagnosis”, “challenge”, “allergy”, “DBPCFC”, “OFC”, “patch”, “CRD”, “component resolved diagnosis”, “hazelnut”, “corylus avellana”, and “tree nut” in the title and abstract, aged 0-18. The Preferred Reporting Items for Systematic Reviews and Meta-Analyses (PRISMA) checklist was used to report this systematic review [23]. We include clinical trials, case-control, and cross-sectional studies. Reviews, discussion papers, editorials, qualitative studies, case reports, case series, conference abstracts and animal studies were excluded. We included studies that presented sufficient data to calculate sensitivity and specificity for at least one allergen component (Cor a 1, Cor a 8, Cor a 9, Cor a 14) (index test). Index tests were sIgE to hazelnut components. All studies were required to have a defined study population, limited to paediatric patients (0–18 years) who were suspected of hazelnut allergies. The reference standard was OFC, open or single-blind or double-blind placebo-controlled food challenge (DBPCFC). Alternatively, the reference standard was a suggestive history of anaphylaxis or serious symptoms due to hazelnut allergy confirmed by an allergist. At least 50% of patients must have performed an oral food challenge.

### 2.1. Study Selection and Data Collection

Two reviewers (M.C. and M.P.) independently screened titles and abstracts and then reviewed the full texts of studies that were considered to potentially meet the inclusion criteria, to identify eligible studies. If a study missed some information necessary to meet the inclusion criteria, authors were contacted. Where we received no response, we used data previously provided by the authors to other reviewers [24]. No language restrictions for included studies were applied: literature in languages other than English has been translated. Data of the following information were extracted: first author, date of publication, country, type of study, sample size, age (0–18 years), gender, and diagnostic tests (sIgE, OFC). Two reviewers (C.C. and M.P.) assessed the quality of the included studies using the Quality Assessment of Diagnostic Accuracy Studies-2 (QUADAS-2) tool [25]. Discrepancies were resolved by discussion and consensus. Measures of diagnostic test accuracy (DTA) and 95% confidence intervals were calculated if they were not reported in the papers.

### 2.2. Statistical Analyses

For each analysis, the cut off threshold was 0.35 kilounits of antibodies per litre (kUA/L). Given the significant heterogeneity found among the results of the included studies, quantified by Chi2, a random-effect meta-analysis model using the DerSimonian-Laird method was run to estimate the pooled test results. The random-effects model was utilized because it considers the risk of significant heterogeneity among studies and gives larger confidence intervals (CIs) than fixed-effect models [26]. Diagnostic odds ratios (DOR), positive and negative likelihood ratios (LR+/LR−), and area under the curve (AUC) were calculated. Fagan nomograms, which consider the LR+ and LR- obtained from the meta-analysis, were also used to estimate the clinical value of the index test [27]. Calculation of post-test probabilities was performed by assuming a pre-test probability that was equal to the prevalence of hazelnut allergies reported in the selected studies. The results were obtained as follows: pretest odds = prevalence/1-prevalence; post-test odds = pretest odds x LR- (LR+); and post-test probability = post-test odds/1+post-test odds. Positive predictive values (PPV) and negative predictive values (NPV) were computed. Publication bias was assessed with the funnel plot proposed by Egger [28]. Statistical analyses were performed using StatsDirect Statistical Software(StatsDirect statistical software. http://www.statsdirect.com. England: StatsDirect Ltd.) and Meta-DiSc Software (Meta-analysis of studies of evaluations of Diagnostic and Screening tests. http://www.hrc.es/investigacion/metadisc_en.htm. Spain: Unit of Clinical Biostatistics team of the Ramón y Cajal Hospital in Madrid.).

## 3. Results

**Figure 1:**
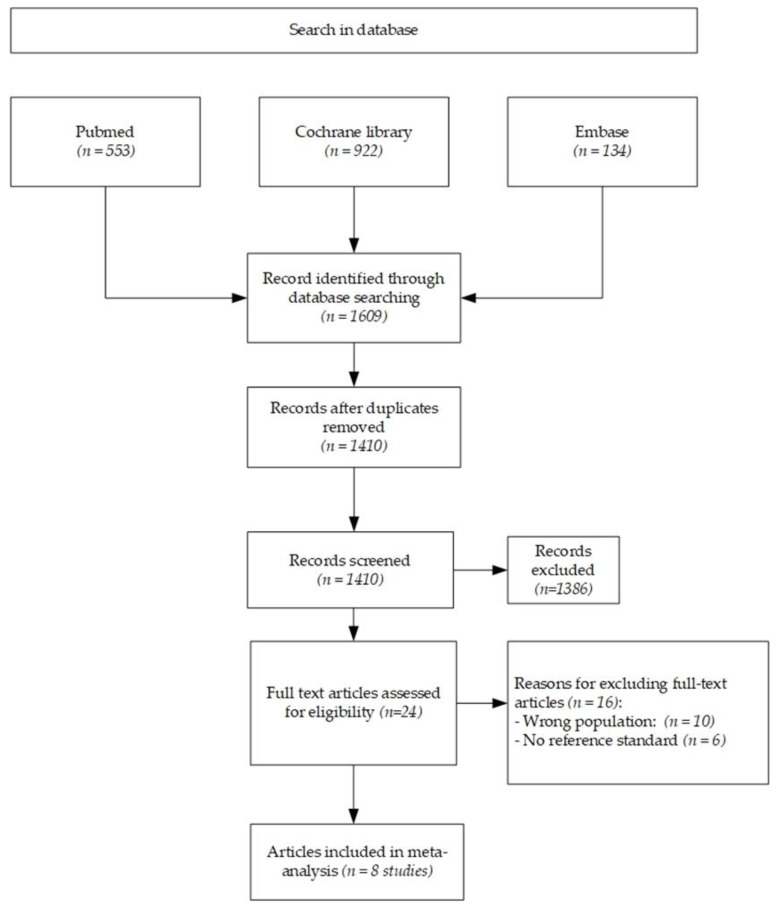
Flow diagram of included studies.

The literature search found 1609 articles. After removing 199 duplicates, 1410 articles were reviewed based on their title and abstract. Among them, 24 full texts were assessed for inclusion, while 1386 articles were excluded based on their title and abstract. Eight studies (Figure 1) that met the research criteria were identified and included in the analysis. All the studies recruited pediatric patients only.

### 3.1. Risk of Bias

**Figure 2:**
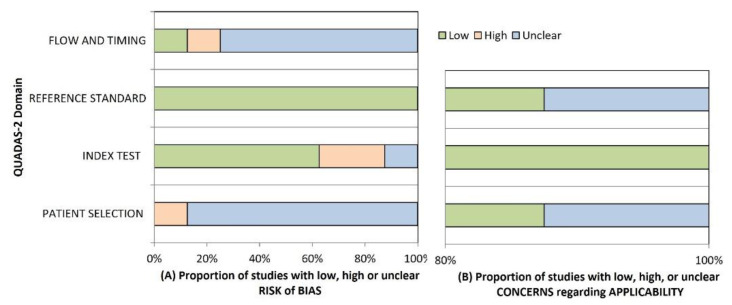
QUADAS-2 Domain. (**A**) Risk of bias; (**B**) Concerns regarding applicability.

Results of the QUADAS-2 tool are reported in Figure 2. One study was found to have high risk of bias (ROB) in the patient selection domain because of case-control design [29]. The remaining studies had an unclear ROB because they failed to meet at least one of the criteria of the domain, mostly the sampling methodology [30,31,32,33,34,35]. There was no concern of ROB for applicability in this domain. In the index test domain, two studies were rated as having high ROB because a pre-specified threshold was not used [31,33]. The remaining studies were ranked as having unclear ROB because it was undetermined whether index test results were interpreted without knowledge of OFC results [29,30,32,34,35]. There was no concern of ROB for applicability. Regarding their reference standard, all studies were scored as having low ROB [29,30,31,32,33,34,35]. There was no concern of ROB for applicability except in one study with unclear ROB in this domain [29]. In flow and timing domains, a study had high ROB because it did not use OFC in all patients and did not include all patients in the analysis [34]. Six studies had unclear ROB because they did not meet a criterion [16,29,30,32,33,35]. One study had low ROB in this domain [31].

### 3.2. Study Characteristics

Extracted data were summarized in Table 1.

Studies were conducted in Europe (*n* = 7), Japan (*n* = 1), and the United States (*n* = 1). We found a total of 757 pediatric cases of suspected hazelnut allergy. All studies measured levels of sIgE to Cor a 1, Cor a 8, Cor a 9, and Cor a 14, using the same assay (ImmunoCAP, ThermoFisher, Uppsala, Sweden). Studies varied at the lower detection limit of hazelnut components between > 0 and 0.35 kilounits of antibody per litre (kUA/L) (Table 1). Regarding inclusion criteria, some studies enrolled children based on clinical history of a suspected hazelnut allergy [16,30,31,33], while others selected children with a clinical impression or convincing history of a hazelnut allergy [34]. A trial investigated children with hazelnut sensitization [35]. Other studies selected children based on the outcome of a food challenge [29] or to determine whether children had reached tolerance to hazelnuts [32]. All studies except one [34] reported the age of children, which ranged from 0.7 to 18 years. Median age varied from a low of 3.4 to 11 years. All papers but one [34] described the gender of recruited children. There were 480 (67%) males. The reference standard was an oral challenge using hazelnuts in all studies. However, OFCs were conducted with different protocols, including open, single blind or double blind. When the OFC was blinded, hazelnut was masked in chocolate products including mousse [16,30], balls [31], pudding [32], bars [33]) or Nutella [34] (Ferrero U.S.A., Inc., Somerset, NJ). In one study [29] the challenge was performed with defatted hazelnut flour for the first 9 doses (blinded) and a portion of 10 hazelnuts for the last dose (unblinded). In one study [35], roasted hazelnuts were used. The outcome of 741 hazelnut challenges was positive in 293 (39%) instances. In 16 patients, the challenge was not performed, and diagnosis was based on clinical history or recent anaphylactic reactions to hazelnuts. 

### 3.3. Diagnostic Accuracy

There was variability in the diagnostic accuracy of sIgE to hazelnut components among studies (Table 2, Table 3 and Table 4).

Overall, both the sensitivity and specificity of IgE to hazelnut components were low (Table 2). The sensitivity and specificity of Cor a 1 sIgE were significantly lower than those of sIgE to both Cor a 9 and to Cor a 14, since a 95% confidence interval (CI) did not overlap. There is only one study by Masthoff et al [29] on Cor a 8 sIgE in children. They found that sIgE to Cor a 8 had a significantly lower sensitivity (5.0 (CI 95%, 0.6–16.9)) than other hazelnut components. The specificity of Cor a 9 sIgE [29] was also significantly lower than that of both Cor a 14 sIgE and Cor a 8 sIgE (95.1 (CI 95%, 83.5–99.4)).

AUC (Table 3) showed that the chance to be able to distinguish between positive and negative Cor a 1 sIgE was only 55%. For Cor a 8 sIgE, it was 58%. The AUCs of Cor a 9 and Cor a 14 were higher, 81% and 87%, respectively, and significantly different from those of Cor a 1 and Cor a 8, as shown by no overlapping 95% CIs. There was no difference between the AUC of Cor a 9 and the AUC of Cor a 14.

Regarding index test predictivity (Table 4), the LR+ of Cor a 1 sIgE was significantly lower than those of sIgE to both Cor a 9 and Cor a 14. Cor a 1 sIgE did not increase the probability of a hazelnut allergy, while sIgE to both Cor a 9 and Cor a 14 slightly increased it. The LR- of Cor a 1 sIgE slightly decreased the probability of having a hazelnut allergy, while both Cor a 9 sIgE and Cor a 14 sIgE moderately decreased it. According to the Fagan nomogram, we fixed the pre-test probability to 39% for hazelnut allergies, which was estimated by the number of children who reacted to hazelnuts in the selected studies. If the test result was positive, the post-test probability of a hazelnut allergy was 34% for Cor a 1 sIgE, 60% for Cor a9 sIgE and 73% for Cor a 14 sIgE. On the other hand, if the test result was negative, the post-test probability of a hazelnut allergy was 55% for Cor a 1 sIgE, 16% for Cor a9 sIgE and 14% for Cor a 14 sIgE.

The DOR (Table 2) of sIgE to Cor a1 was 0.42—lower than that of sIgE to both Cor a 9 and Cor a 14. DOR of Cor a 14 sIgE was not significantly higher than that of Cor a 9 sIgE (18.27 vs. 9.45). Positive predictive value of Cor a 1 sIgE varied from 12% to 46%, Cor a 9 sIgE from 36% to 80% and Cor a 14 sIgE from 61% to 88%. Negative predictive values of sIgE to Cor a 1, Cor a 9 and Cor a 14, respectively, ranged from 25% to 61%, 82% to 100%, and 72% to 100%.

Only one study [33] assessed whether IgE to hazelnut components were associated with the severity of objective symptoms in the hazelnut challenge. They found no correlation between IgE to Cor a 9 and Cor a 14 and the grade of allergic reaction. 

Four of the selected studies considered the diagnostic value of combined IgE to hazelnut components. Beyer [30], Eller [33] and Inoue [35] did not find that the performance of diagnostic tests was improved by combining different components. In contrast, Masthoff [29] reported that combined positive IgE to Cor a 9 and Cor a 14 had a sensitivity that was similar to that of single molecules and a specificity of 98% that was higher than those of Cor a 9 and Cor a 14.

## 4. Discussion

This study has provided an assessment of the diagnostic accuracy of sIgE on individual hazelnut proteins in the diagnostic work-up of hazelnut allergies in children. Available hazelnut component tests include storage proteins Cor a 9 and Cor a 14, PR-10 Cor a 1, and LPT Cor a 8 [22,36,37].

The studies included in the present research had sensitivity and specificity varying from 50% to 70% and 9% to 58%, respectively, for Cor a 1 sIgE; from 74% to 100% and from 56% to 80%, respectively, for Cor a 9 sIgE; and from 69% to 100% and 75% to 88%, respectively, for Cor a 14 sIgE. When we performed an overall estimate of sensitivity and specificity, Cor a 9 sIgE and Cor a 14 sIgE were superior to Cor a 1 sIgE.

AUC that was unaffected by the prevalence of disease, since it was based on combined sensitivity and specificity, showed a moderate diagnostic accuracy for Cor a 1 sIgE and Cor a 8 sIgE. The AUCs of Cor a 9 sIgE and Cor a 14 sIgE were significantly more elevated than those of Cor a 1 sIgE and Cor a 8 sIgE. The AUC of Cor a 9 sIgE was similar to that of Cor a 14 sIgE. It is unclear why Cor a 1 sIgE had a lower sensitivity/specificity. Several hypotheses may be offered. Hazelnut sensitization can be genuine or due to IgE-mediated cross-reactivity to Bet v 1. Children who were primarily sensitized to PR-10 from birch or birch-related tree pollen [38] can have positive Cor a 1-sIgE as the result of a cross-reaction, which may be asymptomatic [39]. Since Cor a 1 is sensitive to gastric digestion and heat-labile, children who are only sensitized to this component often do not develop allergic symptoms. However, the severity of positive OFC on hazelnuts was not associated with positive results for any IgE to hazelnut components. The studies we selected did not allow us to separately assess the diagnostic accuracy of IgE to hazelnut components in children with or without Bet v 1 sensitization. Only three of the selected studies considered hazelnut allergies in children in relation to birch pollen allergies. Buyuktiryaki [32] found that only 4 (7,8%) children with hazelnut allergies were sensitized to tree pollen, and it was not stated how many children were allergic to birch. Eller found that sensitization to Bet v 1 was not associated with hazelnut allergies. Masthoff found that most children with subjective hazelnut allergies were sensitized to birch pollen and Bet v 1. Neither Buyuktiryaki [32], Eller [33] or Masthoff [29] reported levels of IgE to hazelnut allergen components (Cor a1, Cor a8, Cor a9 and Cor a14) in children with birch pollen sensitization compared with those in children who were not sensitized to birch pollen. Other explanations may be the smaller amount of Cor a 1 available compared with other components in fruit with reduced recognition, or less induction of IgE production by Cor a 1. These speculations require confirmation by further studies. There is not sufficient data to consider the specificity and sensitivity of sIgE to Cor a 8 since we have found only one study. Cor a 8 is more difficult to evaluate due to its great variability depending on the geographic area considered. Sensitization to LTP is more common in Mediterranean areas, but its clinical relevance is still debated. Another question is whether combining the results of studies addressing IgE to hazelnut components may improve diagnostic accuracy. Since there are contrasting data on this issue, further studies are necessary.

In clinical practice, it is recommended that children should not avoid hazelnuts without a clear diagnosis. On the other hand, children with hazelnut allergies should be carefully identified since serious reactions may develop following hazelnut ingestion. The gold standard for diagnosing hazelnut allergies is the OFC. However, extensive use of OFC is not economically sustainable. It requires a large amount of healthcare resources, and it is a stressful event both for patients and their caregivers. Moreover, OFC is a diagnostic procedure that involves some risks and requires an appropriate setting with personnel able to manage severe reactions such as anaphylaxis. Therefore, we have assessed whether component-resolved diagnosis for hazelnuts might predict hazelnut allergies and reduce the number of patients who need an OFC. This is of greater importance in the SARS-CoV-2 pandemic context, in which it is necessary to limit hospital tests as much as possible [40,41].

The prevalence of hazelnut allergies varied from 9% to 69% in the populations of the selected studies, and it is higher than in the general population of children who reported a hazelnut allergy in 3% of cases [42]. Higher prevalence of the disease increases PPV and decreases NPV. So, it is better to consider likelihood ratios that are not affected by the prevalence of the disease in the studied population. We calculated the post-test probability by using LRs and Fagan nomograms. We fixed the pre-test probability of a hazelnut allergy to 39%, which corresponds to the number of children with confirmed hazelnut allergies in the selected studies. We have determined that the post-test probability of a positive result was 34% for Cor a 1 sIgE, 60% for Cor a9 sIgE and 73% for Cor a 14 sIgE. Therefore, a positive result of sIgE to hazelnut components is not able to correctly identify children with hazelnut allergies. Negative hazelnut component sIgEs are more able to predict tolerance to hazelnuts. However, the post-test probability of negative result is too high for Cor a9 sIgE (16%), Cor a 14 sIgE (14%), and especially for Cor a 1 sIgE (55%) to reach a distinct diagnosis. 

The results of DOR, which may vary from zero to infinity, are along the same lines. Higher values of DOR indicate a greater chance of a positive result for the index test in a person with a hazelnut allergy, compared with children who tolerate hazelnuts. The DOR of sIgE to Cor a1 was low, while the DORs of sIgE to Cor a 9 and Cor a 14 were significant. The DOR of sIgE to Cor a 1 was lower than that of sIgE to both Cor a 9 and Cor a 14. Children with positive Cor a 14 sIgE were at higher risk of having hazelnut allergies than those with positive Cor a 9 sIgE, but the difference was not significant. To our knowledge, only one systematic review with metanalysis about hazelnut allergy testing has been published until now (24). In agreement with our results, it found that the diagnostic accuracy of sIgE to Cor a 1 was lower than that of Cor a9 sIgE and Cor a 14 sIgE.

The strength of this study is a highly sensitive research strategy performed without language limitations, using various databases that permitted a complete literature review. All the studies included in the metanalysis performed an OFC to reach the diagnosis of a hazelnut allergy in more than 50% of the patients enrolled. A wide number of children were included. The strength of the findings can be limited by differences in inclusion criteria among the selected studies. It was not possible to analyze the diagnostic value of IgE to hazelnut components in studies with similar inclusion criteria since data were insufficient. However, we think that the criteria of our study are large enough to comprise the diversity of studies and the conditions in which the test is used, which also being satisfactorily narrow to obtain important answers when studies are considered together. Another relevant limitation is that if children with a genuine allergy to hazelnuts and those with a birch allergy and cross-reactive allergy to hazelnut are mixed up, the sensitivity and specificity of IgE components are diluted. As a result, the strength of Cor a9 and Cor a14 assessment in genuine hazelnut allergies in children is lost. A weakness may be represented by the limited number of studies retrieved. There is especially a paucity of studies on Cor a 8 sIgE. Another limitation is that there was heterogeneity across the studies on Cor a 1 sIgE. Finally, data were not divided on the basis of other variables, including sex or age, since information was lacking in the included studies.

## 5. Conclusions

Our analysis has shown that an OFC will still, in many cases, be necessary to prove clinical manifestations of hazelnut allergies. Measurement of IgE levels to Cor a 9 and Cor a 14 might have the potential to improve specificity in detecting clinically tolerant children among hazelnut-sensitized ones. This may lead to a reduction in the number of OFCs. Studies on the general population are warranted to elucidate this issue.

## Figures and Tables

**Table 1:** Summary of the included studies. In all studies, specific IgE to Cor a 1, Cor a 8, Cor a 9, and Cor a 14 was measured. DBPCFCF = double-blind placebo-controlled food challenge.

Summary of the Included Studies					
				Oral Food Challenge	
Author, Country, Year	Study Population	Age (years) M/F	Lower limit IgE Positive Test (kUA/L)	Participants (%)	Number Positive (%)
Beyer, Germany, 2015 [30]	143 children with suspected hazelnut allergy	Age (median, quartile) Tolerant 4.7 (2.1–8.1) Allergic 4.3 (2.2–6.1) −98/45	0.10	143 (100%) of which 46/143 (32%) DBPCFCF	99 (69%)
Brandström, Sweden, 2015 [31]	40 children referred for oral challenge for hazelnut allergy suspicion	Age (median, range) 11 (6–18) 23/17	0.10	40 (100%) DBPCFC	8 (20%)
Buyuktiryaki, Turkey, 2016 [32]	64 children with hazelnut allergy to determine resolution of hazelnut allergy	Age (median, interquartile) 3.4 (2.1–7.2) 45/19	0.10	56 (87.5%) DBPCFC 8 not performed because of anaphylaxis within the last 12 months	24 (42%)
Eller, Denmark, 2016 [33]	155 children with suspected hazelnut allergy	Age 5.1 (0.7–15.5) 100/55	0.35	140 (90%) open challenge 15 DBPCFC	65 (41%)
Grabenhenrich, Germany, 2016 [16]	142 children with suspected hazelnut allergy	Age (median, interquartile) 4.5 (2.1–7.6) 97/45	>0	142 (100%) open, single blind, double blind challenge.	44 (31%)
Inoue, Japan, 2019 [35]	91 children sensitized to hazelnut	Age (median, interquartile) 7.3 (5.9–10.5) 63/28	0.35	91 (100%) open food challenge	9 (9%)
Kattan, US, 2014 [34]	33 children with clinical impression of hazelnut allergy 9 children with history of objective symptoms with hazelnut ingestion	-	0.10	33 (78%) open challenge 9 not performed because of a history of objective symptoms with ingestion of hazelnut	4 (12%)
Masthoff, Netherlands, 2013 [29]	81 children Retrospective equally powered groups with positive/negative challenge	Age (median, interquartile) 8 (7–12) 54/27	0.35	81 (100%) DBPCFC	40 (49%)

**Table 2:** Diagnostic accuracy of sIgE to Cor a1, Cor a9, Cor a14. DOR = diagnostic odd ratio.

Author	Sensitivity (%)	(95%CI)	Specificity (%)	(95%CI)	DOR	(95%CI)
Cor a 1						
Brandström [31]	50	(5.7–84.4)	12.5	(3.5–29.0)	0.14	(0.03–0.81)
Eller [33]	49.2	(36.6–61.9)	58.9	(48.0–69.2)	1.39	(0.73–2.64)
Masthoff [29]	70.0	(53.5–83.4)	9.8	(2.7–23.1)	0.25	(0.07–0.87)
Pooled	56.6	(47.0–65.9)	37.4	(30.0–45.3)	0.42	(0.09–1.89)
Heterogeneity, Chi2	4.60 *p* = 0.100		43.3 *p* = 0.000		9.9 *p* = 0.007	
Cor a 9						
Brandstrom [31]	100	(63.1–100)	56.3	(37.7–73.6)	21.69	(1.15–407.76)
Eller [33]	74.2	(61.5–84.0)	67.9	(57.1–77.3)	5.94	(2.93–12.06)
Kattan [34]	84.6	(54.6–98.1)	65.5	(45.7–82.1)	10.45	(1.93–56.64)
Masthoff [29]	83.0	(67.2–92.7)	80.0	(65.1–91.2)	19.43	(6.32–59.75)
Pooled	79.5	(71.5–86.2)	68.1	(60.9–74.6)	9.45	(4.92–18.13)
Heterogeneity, Chi2	5.4 *p* = 0.145		4.9 *p* = 0.180		3.5 *p* = 0.320	
Cor a 14						
Beyer [30]	84.1	(69.9–93.4)	80.8	(71.7–88.0)	22.26	(8.61–57.56)
Brandstrom [31]	100	(63.1–100)	84.6	(67.2–94.7)	85.00	(4.25–1699.61)
Buyuktiryaki [32]	84.6	(65.1–95.6)	88.0	(68.8–97.5)	49.00	(11.14–215.60)
Eller [33]	80	(68.2–88.9)	84.4	(75.3–91.2)	21.71	(9.44–49.96)
Kattan [34]	69.2	(38.6–90.9)	82.8	(64.2–94.2)	10.80	(2.36–49.46)
Masthoff [29]	70	(53.85–83.4)	75.6	(59.7–87.6)	7.23	(2.71–19.32)
Pooled	80.2	(74.0–85.5)	82.4	(77.7–86.4)	18.27	(10.24–32.59)
Heterogeneity, Chi2	8.4 *p* = 0.135		2.35 *p* = 0.799		6.92 *p* = 0.227	

**Table 3:** Area under the curve (AUC) of sIgE to hazelnut components.

Author	AUC	95%CI
**Cor a1** Masthoff [29]	0.43	0.3–0.55
Beyer [30]	0.56	0.46–0.66
Grabenhenrich [16]	0.55	0.46–0.65
Inoue [35]	0.72	0.55–0.9
Pooled	0.55	0.46–0.64
**Cor a8** Masthoff [29]	0.51	0.39–0.64
Beyer [30]	0.63	0.53–0.73
Grabenhenrich [16]	0.62	0.52–0.72
Inoue [35]	0.58	0.39–0.78
Pooled	0.59	0.54–0.65
**Cor a9** Masthoff [29]	0.87	0.79–0.96
Beyer [30]	0.8	0.72–0.88
Eller [33]	0.78	0.7–0.85
Grabenhenrich [16]	0.8	0.72–0.88
Inoue [35]	0.71	0.52–0.89
Pooled	0.81	0.77–0.84
**Cor a14** Masthoff [29]	0.8	0.7–0.9
Beyer [30]	0.89	0.83–0.95
Eller [33]	0.85	0.77–0.94
Grabenhenrich [16]	0.89	0.83–0.95
Buyuktiryaki [32]	0.93	0.85–1
Inoue [35]	0.65	0.44–0.86
Pooled	0.87	0.82–0.92

**Table 4:** Positive predictive value (PPV), negative predictive value (NPV), likelihood ratio (LR) of IgE to Cor a1, Cor a9, Cor a14.

	PPV (%)	(95%CI)	NPV (%)	(95%CI)	LR+	(95%CI)	LR−	(95%CI)
**Cor a 1**								
Brandström [31]	12.5	(1–24)	50.0	(15.4–84.6)	0.57	(0.28–1.16)	4.0	(1.27–2.62)
Eller [33]	46.4	(34.6–58.1)	61.6	(51.4–71.9)	1.2	(0.84–1.7)	0.86	(0.64–1.16)
Masthoff [26]	43.1	(31–55.1)	25	(3.8–46.2)	0.78	(0.62–0.97)	3.08	(1.08–8.74)
Pooled					0.85	(0.58–1.26)	1.99	(0.63–6.21)
Heterogeneity, Chi2					6.0 *p* = 0.050		11.5 *p* = 0.003	
**Cor a 9**								
Brandstrom [31]	36.4	(16.3–56.5)	100	(100–100)	2.15	(1.42–3.26)	0.1	(0.07–1.5)
Eller [33]	62.3	(51.5–73.2)	78.2	(69–84.4)	2.29	(1.64–3.2)	0.39	(0.25–0.60)
Kattan [34]	52.4	(31–73.7)	90.5	(77.9–100)	2.45	(1.41–4.26)	0.24	(0.06–0.86)
Masthoff [29]	80.5	(68.4–2.6)	82.5	(70.7–94.3)	4.16	(2.20–7.83)	0.21	(0.11–0.43)
Pooled					2.47	(1.93–3.17)	0.31	(0.21–0.45)
Heterogeneity, Chi2					3.6 *p* = 0.309		3.1 *p* = 0.377	
**Cor a 14**								
Beyer [30]	66.1	(53.7–78.5)	92	(86.2–97.7)	4.38	(2.87–6.70)	0.20	(0.1–0.39)
Brandstrom [31]	61.5	(35.1–88)	100	(100–100)	5.67	(2.60–12.35)	0,07	(0–1)
Buyuktiryaki [32]	88	(75.3–100)	84.6	(70.7–98.5)	7.00	(2.77–17.67)	0.14	(0.06–0.36)
Eller [33]	78.8	(68.9–88.7)	85.4	(78.1–92.7)	5.14	(3.13–8.45)	0.24	(0.14–0.39)
Kattan [34]	64.3	(39.2–89.4)	85.7	(72.8–98.7)	4.02	(1.67–9.64)	0.37	(0.16–0.85)
Masthoff [29]	73.7	(59.7–87.7)	72.1	(58.7–85.5)	2.87	(1.61–5.1)	0.40	(0.24–0.66)
Pooled					4.44	(3.48–5.67)	0.26	(0.18–0.37)
Heterogeneity, Chi2					3.9 *p* = 0.560		7.4 *p* =0.196	

## Data Availability

Data sharing is not applicable to this article.
